# Deep learning evaluation of echocardiograms to identify occult atrial fibrillation

**DOI:** 10.1038/s41746-024-01090-z

**Published:** 2024-04-13

**Authors:** Neal Yuan, Nathan R. Stein, Grant Duffy, Roopinder K. Sandhu, Sumeet S. Chugh, Peng-Sheng Chen, Carine Rosenberg, Christine M. Albert, Susan Cheng, Robert J. Siegel, David Ouyang

**Affiliations:** 1 School of Medicine, University of California, San Francisco, CA; Division of Cardiology, San Francisco Veterans Affairs Medical Center, San Francisco, CA USA; 2 Cedars-Sinai Smidt Heart Institute, Los Angeles, CA USA

**Keywords:** Atrial fibrillation, Echocardiography

## Abstract

Atrial fibrillation (AF) often escapes detection, given its frequent paroxysmal and asymptomatic presentation. Deep learning of transthoracic echocardiograms (TTEs), which have structural information, could help identify occult AF. We created a two-stage deep learning algorithm using a video-based convolutional neural network model that (1) distinguished whether TTEs were in sinus rhythm or AF and then (2) predicted which of the TTEs in sinus rhythm were in patients who had experienced AF within 90 days. Our model, trained on 111,319 TTE videos, distinguished TTEs in AF from those in sinus rhythm with high accuracy in a held-out test cohort (AUC 0.96 (0.95–0.96), AUPRC 0.91 (0.90–0.92)). Among TTEs in sinus rhythm, the model predicted the presence of concurrent paroxysmal AF (AUC 0.74 (0.71–0.77), AUPRC 0.19 (0.16–0.23)). Model discrimination remained similar in an external cohort of 10,203 TTEs (AUC of 0.69 (0.67–0.70), AUPRC 0.34 (0.31–0.36)). Performance held across patients who were women (AUC 0.76 (0.72–0.81)), older than 65 years (0.73 (0.69–0.76)), or had a CHA_2_DS_2_VASc ≥2 (0.73 (0.79–0.77)). The model performed better than using clinical risk factors (AUC 0.64 (0.62–0.67)), TTE measurements (0.64 (0.62–0.67)), left atrial size (0.63 (0.62–0.64)), or CHA_2_DS_2_VASc (0.61 (0.60–0.62)). An ensemble model in a cohort subset combining the TTE model with an electrocardiogram (ECGs) deep learning model performed better than using the ECG model alone (AUC 0.81 vs. 0.79, p = 0.01). Deep learning using TTEs can predict patients with active or occult AF and could be used for opportunistic AF screening that could lead to earlier treatment.

## Introduction

Atrial fibrillation (AF) is the most common cardiac arrhythmia and is associated with significant morbidity and mortality^1,2^. AF is frequently paroxysmal and asymptomatic, and therefore, undetected until it becomes symptomatic or presents with complications such as stroke or heart failure^3^. Given the potential promise of intervening in early AF with anticoagulation to reduce stroke risk or therapies to maintain sinus rhythm, a number of trials have investigated the utility of routine screening for AF using either home or office-based intermittent and/or continuous electrocardiography (ECG)^4–10^. These studies reveal that many cases of AF are not detected with conventional screening practices.

Recent work has shown that artificial intelligence (AI) applied to ECGs can predict concurrent paroxysmal AF as well as incident AF from sinus ECGs^11–13^. This work has even been validated in prospective clinical trials, suggesting the value and efficacy of opportunistic screening by AI^14^. Transthoracic echocardiograms (TTEs) are routinely obtained in patients with cardiovascular symptoms and individuals who are at high risk for AF. As the most common cardiovascular imaging modality, TTEs may provide additional structural information complementary to ECGs that could also be opportunistically used to help identify occult AF. AI is increasingly being applied to TTEs for image acquisition, image interpretation, and as a diagnostic and prognostic tool^15^. We have previously shown that video-based AI can determine left atrial and ventricular sizes, the presence of pacemaker leads, as well as determine ejection fraction with better accuracy than human experts^16,17^. AI interpretation of echocardiograms can also predict clinical disease processes^18,19^. In this study, we sought to determine whether a deep learning model using echocardiogram videos could identify patients in AF, including even those in sinus rhythm at the time of the echocardiogram.

## Results

### Patient characteristics

**Fig. 1:**
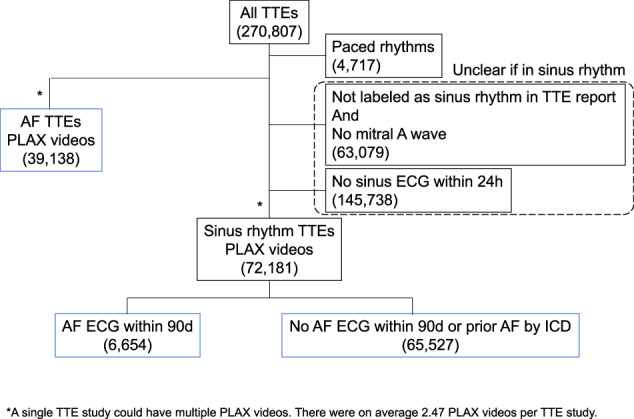
Cohort diagram showing included and excluded TTE studies.

Our cohort included 111,319 TTEs, of which 39,138 studies were in AF and 72,181 studies were in sinus rhythm (Fig. 1). Among those in sinus rhythm, 6,654 studies were in patients with concurrent paroxysmal AF, while 65,527 studies were in patients without evidence of concurrent AF. Patients with atrial fibrillation were on average older (75.2 vs. 66.0 years old), less often female (40.1 vs. 45.5%), more often White (67.9 vs. 57.1%), and had more comorbidities (Table 1). The average left atrium area was larger (26.0 vs. 19.3 cm^2^), and the CHA_2_DS_2_VASc score was marginally higher (3.9 vs. 3.1). Among those in sinus rhythm, patients with concurrent paroxysmal AF were also older (72.1 vs. 65.3 years old), less often female (39.93 vs. 45.9%), more often white (64.2 vs. 56.4%), and had more comorbidities. The left atrial area was slightly larger (21.8 vs. 19.1 cm^2^), and the CHA_2_DS_2_VASc score was higher (3.8 vs. 3.0).

**Table 1:** Demographics and clinical characteristics of patients with TTEs in AF, sinus rhythm with or without concurrent AF within 90 days

	AF	Sinus	*p* value	Sinus with AF ± 90d	Sinus without AF ± 90d	*p* value
*n*	39,138	72,181		6654	65,527	
PLAX videos per patient (SD)	3.61 (2.9)	3.09 (2.4)	<0.001	3.09 (2.3)	3.07 (2.4)	0.910
Age (years) (SD)	75.2 (13.1)	66.0 (16.9)	<0.001	72.1 (13.6)	65.3 (17.1)	<0.001
Female (%)	15694 (40.1)	32755 (45.4)	<0.001	2657 (39.9)	30098 (45.9)	<0.001
Race (%)			<0.001			<0.001
American Indian	80 (0.2)	155 (0.2)		10 (0.2)	145 (0.2)	
Asian	2539 (6.5)	5100 (7.1)		547 (8.2)	4553 (7.0)	
Black	4258 (10.9)	11,746 (16.3)		759 (11.4)	10,987 (16.8)	
Hispanic	2845 (7.3)	7462 (10.4)		531 (8.0)	6931 (10.6)	
White	26,525 (67.9)	41,088 (57.1)		4270 (64.2)	36,818 (56.4)	
Other	2170 (5.6)	5265 (7.3)		370 (5.6)	4895 (7.5)	
Pacific Islander	72 (0.2)	180 (0.3)		15 (0.2)	165 (0.3)	
Unknown	552 (1.4)	959 (1.3)		145 (2.2)	814 (1.2)	
Heart Failure (%)	18,697 (47.8)	19,574 (27.1)	<0.001	2865 (43.1)	16,709 (25.5)	<0.001
Hypertension (%)	24,881 (63.6)	39101 (54.2)	<0.001	4163 (62.6)	34,938 (53.3)	<0.001
Prior CVA (%)	10,816 (27.6)	18,150 (25.1)	<0.001	2045 (30.7)	16,105 (24.6)	<0.001
Prior Myocardial Infarction (%)	4757 (12.2)	8443 (11.7)	0.025	1155 (17.4)	7288 (11.1)	<0.001
Peripheral Artery Disease (%)	6378 (16.3)	7560 (10.5)	<0.001	1183 (17.8)	6377 (9.7)	<0.001
Diabetes (%)	9391 (24.0)	16507 (22.9)	<0.001	1789 (26.9)	14,718 (22.5)	<0.001
Smoker (%)	170 (11.5)	168.2 (11.1)	<0.001	273 (4.1)	2820 (4.3)	0.460
Height (cm) (SD)	79.73 (22.3)	78.2 (21.1)	<0.001	169 (11.2)	168.2 (11.1)	<0.001
Weight (kg) (SD)	1752 (4.5)	3093 (4.3)	0.139	78.4 (20.2)	78.23 (21.2)	0.631
LA Area (cm^2^) (SD)	26.0 (8.2)	19.3 (5.7)	<0.001	21.8 (6.4)	19.07 (5.6)	<0.001
LV Ejection Fraction (%) (SD)	52.6 (16.1)	59.1 (13.4)	<0.001	55.97 (15.4)	59.4 (13.1)	<0.001
CHA_2_DS_2_VASc (SD)	3.9 (2.1)	3.1 (2.1)	<0.001	3.84 (2.1)	3.0 (2.1)	<0.001

### Deep learning model performance

**Fig. 2:**
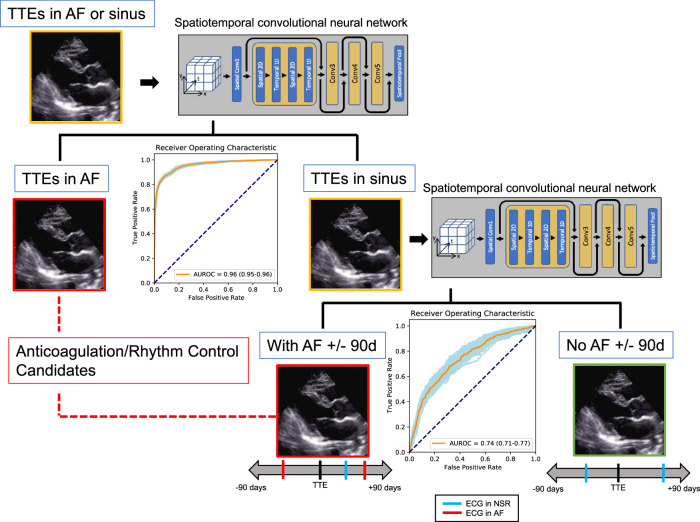
Diagram and performance of the deep learning-based algorithm for identifying patients with active AF or paroxysmal AF at the time of TTE. Using two serial convolutional neural networks, TTEs were classified as being in active AF or in sinus with paroxysmal AF within 90 days, both scenarios where anticoagulation or rhythm control would be potentially indicated. TTEs were first stratified as being in AF or sinus rhythm. The model first determined AF versus sinus rhythm with an AUROC of 0.96 (95% CI 0.95–0.96). From the subset in the sinus, the model then further predicted which TTEs had concurrent paroxysmal AF, defined as having AF on ECG within 90 days before or after, with an AUROC of 0.74 (0.71–0.77).

When tested on a held-out dataset, our deep learning model distinguished whether a TTE was in AF or sinus rhythm with an AUC of 0.96 (0.95–0.96) and an AUPRC of 0.91 (0.90–0.92) (Fig. 2). Among those TTEs in sinus rhythm, the model predicted concurrent paroxysmal AF with an AUC of 0.74 (0.71–0.77) and an AUPRC of 0.19 (0.16–0.23). At the Youden index, the sensitivity was 0.69, specificity 0.68, accuracy 0.68, PPV 0.17, NPV 0.96, and F1 score 0.28. When extending the window for AF detection from 90 to 365 days, the model performance remained similar (AUC 0.71 (0.69–0.74), AUPRC 0.31 (0.27–0.36)) (Supplementary Fig. 1). When applied to the external site test dataset of 10,203 TTEs, the model achieved an AUC of 0.69 (0.67–0.70) and an AUPRC of 0.34 (0.31–0.36) in discriminating patients with a history of AF (Supplementary Fig. 2).

The model also performed similarly in multiple subgroup analyses. In women, the AUC was 0.76 (0.72–0.81). In higher-risk patients (age ≥65 or CHA_2_DS_2_VASc ≥2), the AUC was 0.73 (0.69–0.76) and 0.73 (0.79–0.77). In patients <65 years old, the AUC was 0.75 (0.69–0.81), and in non-white patients, the AUC was 0.73 (0.68–0.78).

### Comparison to current AF risk prediction methods

**Fig. 3:**
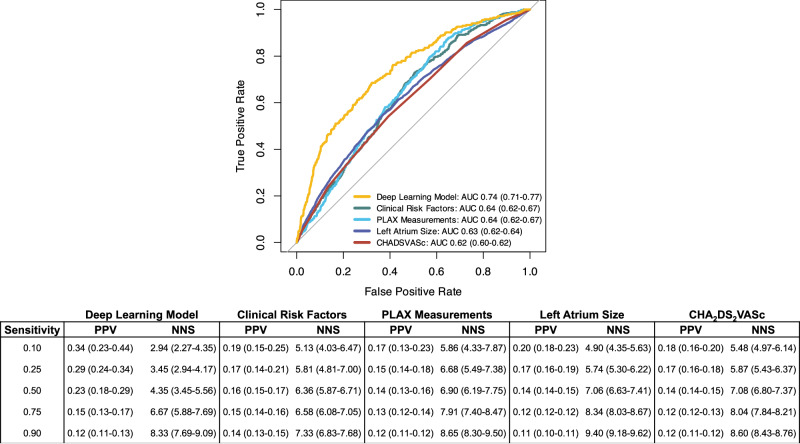
AF prediction performance using deep learning compared to other risk prediction methods. Performance of a deep learning model for prediction of concurrent paroxysmal AF from PLAX TTE videos in sinus rhythm compared to models using CHARGE-AF clinical risk factors (age, race, height, weight, hypertension, smoking history, diabetes, heart failure, and myocardial infarction history), PLAX measurements (age, sex, LA diameter, LV end-diastolic and systolic diameters, septal diameter, LV posterior wall diameter), left atrial size, and CHA_2_DS_2_VASc score. Model positive predictive value (PPV) and number needed to screen (NNS) with 95% confidence intervals at different sensitivity thresholds are presented.

When compared to other AF risk prediction methods, the deep learning model performed better than predicting concurrent paroxysmal AF using CHARGE-AF clinical risk factors (AUC 0.64 (0.62–0.67)), PLAX measurements (AUC 0.64 (0.62–0.67)), LA size (AUC 0.63 (0.62–0.64)), or CHA_2_DS_2_VASc score (AUC 0.62 (0.60–0.62)) (Fig. 3)^20^. Model performance was also compared across different sensitivity thresholds in our cohort (Fig. 3). At a sensitivity of 0.50, the number of patients deemed high risk that would need to be screened to detect one true case of AF was 4.35 (5.56–3.45) using the deep learning model compared to 6.36 (5.87–6.71) using a clinical risk regression model, 6.90 (6.19–7.75) by PLAX measurements, 7.06 (6.63–7.41) by LA Area, and 7.08 (6.80–7.37) using the CHA_2_DS_2_VASc score.

In a small exploratory prospective analysis of 32 patients with a history of paroxysmal AF who had continuous remote telemetry monitoring but seemingly structurally normal heart (normal left atrium size and left ventricular ejection fraction), we found that the model was still able to predict which patients would have significant AF defined as a burden more than 6 min, 1.5, or 24 h with AUCs of 0.69 (0.50–0.88), 0.71 (0.53–0.90), and 0.70 (0.45–0.94).

Given robust performance of prior models in predicting paroxysmal AF using sinus rhythm ECGs, we created an ensemble model and applied it to a subset of 2411 TTEs paired with contemporaneous 12-lead sinus rhythm ECGs. We used linear regression to derive the optimal weighted contributions of ECG and TTE model predictions. The ensemble model weights were 0.35 and 0.48 for the ECG and TTE predictions, respectively, indicating that the TTE predictions contributed equally, if not more heavily, to the final combined ensemble prediction. The ensemble model demonstrated small but significant increases in performance (AUC 0.81 (0.78–0.84)) compared to either ECG or TTE-based models alone (AUC 0.79 (0.76–0.82), DeLong’s test *p* = 0.01; AUC 0.73 (0.70–0.77), *p* < 0.01; respectively) (Supplementary Fig. 3).

Lastly, we conducted model interpretability analyses to better visualize potential areas of focus of our deep learning model. While there were some potential patterns highlighting areas around the left atrium and mitral valve, we did not feel that this signal was consistent. Representative images are provided in the Supplement (Supplementary Fig. 4).

## Discussion

In this study, we found that a deep learning model based on echocardiographic videos could distinguish a high-risk patient group by identifying both patients in AF as well as those in sinus rhythm with concurrent paroxysmal AF. The model outperformed the prediction of concurrent paroxysmal AF using CHARGE-AF risk factors, PLAX measurements, left atrial size, or CHA_2_DS_2_VASc score. While the accuracy for predicting concurrent AF was moderate, applying such a model to routinely acquired TTEs and potentially to bedside TTEs at the point-of-care could present opportunities for improving future efforts to screen for AF and prevent its complications.

AF is frequently asymptomatic and therefore goes unidentified until complications such as a stroke occur^21^. In fact, occult AF is detected in up to 20% of patients with acute stroke^22,23^. Being able to identify patients with occult AF in patients before or after a stroke is clinically important as initiation of anticoagulation reduces the risk of stroke in atrial fibrillation^24^. Additionally, earlier identification of occult AF can allow initiation of rhythm control strategies, which may have more beneficial long-term outcomes^25–27^.

Echocardiography is a noninvasive test frequently performed in patients who are at risk for unrecognized paroxysmal AF whether for direct evaluation of the etiology of thromboembolism or stroke or for other cardiac pathologies that are correlated with high AF risk^28^. In our study, we found that TTE videos are able to capture some of the structural changes in the heart that may signal ongoing atrial fibrillation, even when the heart is in sinus rhythm. Echocardiographic parameters, including left atrial size and function, left ventricular wall thickness, diastolic function, LAVI/a’ (ratio of LA volume index to tissue Doppler A’), and septal PA-TDI (atrial conduction time) have been used to identify patients with AF^29–36^. However, all of these assessments require specific measurements to be performed at the time of the TTE and may not be measurable in all patients. A strength of our model is that it only requires a single PLAX video clip, as opposed to a whole echocardiogram, and does not require any Doppler data. The PLAX is a highly standardized and routinely obtained view, even by novice scanners. By requiring only a short video clip from the PLAX view, our model is an efficient and pragmatic approach that could be implemented in real-time clinical practice.

Our model performed well in identifying active AF during the time of TTE. Though AF can be routinely detected by ECG and physical exam, ECG tracings may not always be available, and other irregular rhythms appreciated by the exam could be due to ectopic beats and conduction abnormalities that can mimic AF. Automated rhythm identification may be especially useful in situations where TTE images are acquired without concomitant ECG information, such as during the performance of point-of-care ultrasound (POCUS).

The identification of patients with paroxysmal AF at the time of a sinus rhythm TTE is more challenging. Nevertheless, our model was able to identify concurrent paroxysmal AF within 90 days with an accuracy that is clinically meaningful. Using the TTE-based deep learning model, the number needed to screen to detect one true case of AF could be as low as three patients at a sensitivity threshold of 0.10 or eight patients at a sensitivity of 0.9 in a high AF prevalence cohort. This performance was better than current widely employed risk stratification methods, including using TTE measurements or clinical risk factors such as the CHA_2_DS_2_VASc or CHARGE-AF scores, all of which, on average, would require screening several more individuals to potentially detect the same number of AF cases. The deep learning model would also be potentially easier to use since it can automatically calculate risk without requiring a provider to measure or input risk factors that may not always be available or reliable in the records or by patient history. We also showed in a small prospective cohort of patients with both normal atrial size and left ventricular ejection fraction, that even in the absence of structural abnormalities classically associated with AF, the model could potentially predict significant AF burden. These additional results would corroborate the theory that higher AF burden is associated with subtle structural changes prior to overt atrial dilation that may also be present over a long time horizon. A current application of our model may be as a first pass screening mechanism to inform additional testing. One could imagine that patients with a positive AF screen from their TTE could trigger an informal chart review or provider visit with potential considerations for more intensive continuous rhythm monitoring.

It should be recognized that our model performed less well than previously published deep learning models using ECG data^11–13^. This suggests that structure alone is likely insufficient in determining concurrent AF, consistent with there being changes in the electrical system of the LA that precede the structural changes seen on imaging. Indeed, previous work has shown that analysis of P wave morphologies and dispersion and atrial premature complexes can predict AF^37–39^. Nevertheless, even in a potential future world where ECG-based deep learning models are widely employed, we believe that there remain compelling clinical applications for using TTE-based prediction models. Since TTEs can often be obtained without a concomitant 12-lead ECG, especially in outpatient settings, a TTE-based deep learning model could be opportunistically integrated into TTE interpretation and provide additional touch points for AF screening that decrease the chance that AF goes undetected between ECGs. This may be especially true as POCUS becomes further included in routine physical exams, which will increase the frequency that TTE views are obtained. Due to the interplay between atrial electrical activity and structure, as LA enlargement can beget AF and also be caused by longstanding AF, we also believe that there is a role for deep learning of TTEs to supplement ECG-based models with joint predictions improving overall AF screening. We showed that an ensemble of predictions from both our TTE-based model as well as our previously published ECG-based deep learning model performed better than using either model alone. A single model using both data types as inputs remains a promising topic for future research.

There are several study limitations to consider. While the prevalence of concurrent paroxysmal AF was similar to other studies at academic centers, the prevalence of AF in our cohort was particularly high due to our study’s design^12,13^. We required that sinus rhythm TTEs have both a TTE in sinus rhythm by TTE report and an ECG within 24 h showing sinus rhythm, which reduced the number of sinus TTEs in our cohort. This therefore increased the relative prevalence of AF TTEs. While the higher proportion of AF cases does not reflect prevalence in the general population, we believe that rigorously defining TTEs in sinus rhythm increased the quality of our model training and, therefore, validity. It should be acknowledged, though, that the number needed to screen would likely be higher than what is reported in our results if the model were to be deployed in a lower AF risk group. Requiring a sinus ECG within 24 h of TTEs in our cohort could also introduce an indication bias for the TTEs included. However, given the general frequency with which ECGs are obtained, we believe that significant bias is less likely. Our model was trained on data from a single center. However, generalizability was demonstrated in a sizable cohort from a separate medical system and across several patient subgroups. For this external cohort, we did not have data to know exactly how long the window of time was between AF diagnosis and TTE, so the prediction task was slightly different and perhaps more difficult. In order to verify the performance and utility of our model, further prospective analyses are needed. Lastly, as with all deep learning models, explainability remains challenging, and we were unable to demonstrate a clear visual representation of the model’s focus despite using state-of-the-art interpretability methods. This may be because, in contrast to image-based models, a video-based convolutional neural network incorporates information from multiple frames, including differential changes from frame to frame that might not be well visualized on a single image. However, the model’s ability to show consistent performance across cohorts and subgroups is again reassuring as to the model’s generalizability.

In conclusion, a deep learning model determined whether a TTE was in sinus rhythm or atrial fibrillation with high accuracy and was able to predict the presence of concurrent paroxysmal atrial fibrillation from sinus TTEs moderately well. The model performed better than what could be predicted from clinical variables or TTE measurements. Thus, deep learning by TTE may offer additional opportunities to guide patient screening for occult atrial fibrillation by identifying patients who may benefit from more intensive monitoring.

## Methods

### Dataset

We identified all TTEs performed between 6/2004 and 6/2021 at Cedars-Sinai Medical Center. We included only those TTEs that were in AF (atrial fibrillation or atrial flutter) or sinus rhythm per the TTE report and by the absence or presence of an identified mitral A wave Doppler velocity. In order to improve label accuracy, TTEs in sinus rhythm were additionally required to have a sinus rhythm ECG within 24 h (Fig. 1). Among those TTEs in sinus rhythm, we further classified concurrent paroxysmal AF cases as patients who had a TTE in sinus rhythm with AF documented on ECG within 90 days before or after the TTE. Control patients had a sinus rhythm TTE and no documented AF within 90 days by ECG and no AF by ICD (International Classification of Diseases) diagnosis prior to or up to 90 days after the TTE (Fig. 2). The 90-day interval was chosen as a cutoff, since we hypothesized that patients who were having paroxysmal AF would likely already demonstrate structural changes on TTE within this period. We performed additional modeling with a 365-day window (before and after TTE) to further explore whether TTE changes could be detected out to 1 year.

Patient characteristics and comorbidities were derived from electronic health records using Elixhauser ICD-10 comorbidity definitions to calculate the CHA_2_DS_2_VASc score (Supplementary Table 1)^40–43^. We used the ICD-10 code I48 to identify AF. The left atrial (LA) area was measured from the apical-4-chamber view and was obtained from the TTE database. LA volumes were not measured at our institution.

For external site validation, we included a cohort of 10,203 TTEs with PLAX views from the publicly available Stanford EchoNet-LVH dataset^18^. This dataset includes TTEs from individuals who underwent imaging as part of routine clinical care at Stanford. Each PLAX video was labeled as having had a prior history of AF or not, which was determined by ICD coding. Videos did not have labels as to whether they were in sinus rhythm or AF at the time of the study and also did not have accompanying ECG tracings. However, an echocardiography level 3 certified cardiologist visually examined the ventricular motion and mitral valve opening patterns in 50 randomly selected TTEs and found that 46 of them (92%) were in sinus rhythm with the other four being in an irregular rhythm of unknown type.

The study was approved by the IRB at Cedars-Sinai Medical Center. This study complied with all IRB ethical regulations and was granted a waiver for written informed consent, given the large-scale and deidentified nature of the data.

### Data preprocessing

TTEs were acquired using Philips EPIQ 7 or iE33 ultrasound machines. For deep learning, each TTE study was initially sourced in Digital Imaging and Communications in Medicine (DICOM) format and contained multiple video loops. We isolated PLAX videos because the PLAX view is present in most TTE studies and captures data on left ventricular function, mitral valve disease, as well as left atrial area. The PLAX view is also readily obtainable and one of the first views visualized during point-of-care ultrasound studies. An automated preprocessing workflow was used to remove identifying information and eliminate unintended human labels. Each subsequent video was cropped and masked to remove text, ECG and respirometer information, and other information outside of the scanning sector. The resulting square images were either 600 × 600 or 768 × 768 pixels depending on the ultrasound machine, and downsampled by cubic interpolation into standardized 112 × 112 pixel videos. Videos were spot-checked to verify view classification and ensure videos with color Doppler were excluded^17^.

For model training and validation, the Cedars-Sinai TTE dataset was split using 80% of TTEs for model training, 10% for validation, and 10% for hold-out testing. Multiple PLAX videos from the same patient TTE study could be used for model training, given prior research showing that this can improve model accuracy^12^. For the test cohort, the final prediction for a TTE study was calculated as the mean of the predictions for individual PLAX videos coming from the study. The Stanford TTE dataset was not used in model training and was used for external testing only.

### Deep learning model selection and training

We trained a convolutional neural network R2 + 1D architecture to determine whether a TTE was in sinus rhythm or AF, and subsequently to predict concurrent paroxysmal AF. The R2 + 1D architecture approximates 3D convolution by using blocks of alternating 2D spatial convolution and 1D temporal convolution with residual connections and spatiotemporal pooling in order to make a single prediction from a whole video clip (Fig. 2)^44^. This video-based convolutional neural network model has been previously successfully employed to make predictions from TTE videos^17,19^. We initialized this model using pretrained weights from the EchoNet-Dynamic dataset. Models were trained to minimize the squared loss between the predicted risk and the actual label (0 for no AF and 1 for AF) with an Adam optimizer set to a learning rate of 0.001. We used a batch size of 64 across 50 epochs. The model was fed video clips of 32 frames created by sampling every other frame. All model training was done using the Python library PyTorch.

### Model performance

For model testing purposes, we tested each stage of the model (1) Determining whether a TTE was in sinus rhythm or AF and (2) Predicting whether a TTE in sinus rhythm was from a patient with ongoing paroxysmal AF) separately on the internal held-out test dataset. Given that the external Stanford dataset was predominantly in sinus rhythm and did not have labels for AF at the time of the study, we tested only the second stage of the model (i.e., predicting concurrent paroxysmal AF), arguably the more difficult prediction task, on this dataset.

Model weights from the epoch with the best validation loss were used for final model performance testing on the held-out dataset as well as the external site test dataset. We created receiver operating characteristic (ROC) and precision-recall (PR) curves to show model performance across different classification thresholds. The overall model performance was summarized using the area under the curve (AUC) for the ROC curve as well as the area under the precision-recall curve (AUPRC). We calculated sensitivity (recall), specificity, accuracy, positive predictive value (PPV, precision), negative predictive value (NPV), and F1 score at the Youden index defined as the threshold with the maximum value for sensitivity+specificity-1. We report confidence intervals using 1000 bootstrapped samples.

We compared our deep learning model to AF prediction by logistic regression using all clinical variables that make up the CHARGE-AF prediction score that were available^20^. The CHARGE-AF risk model is one of the most widely utilized clinical risk models for AF prediction and includes age, race, height, weight, systolic and diastolic blood pressure, smoking history, anti-hypertension medication use, diabetes, heart failure, and myocardial infarction history. Of these, the only variables not available in our database were blood pressure and anti-hypertension medication use at the time of the TTE study. We included the history of hypertension instead to help capture similar risk information. Our final clinical risk model included age, race, height, weight, hypertension, smoking history, diabetes, heart failure, and myocardial infarction history. We additionally compared our deep learning model to AF prediction by models using PLAX measurements (age, sex, left atrium diameter, left ventricle end-diastolic diameter, left ventricle end-systolic diameter, interventricular septum diameter, and left ventricle posterior wall diameter), CHA_2_DS_2_VASc score, and left atrial (LA) area on TTE. These statistical analyses were conducted using R software (version 3.4.1, Vienna, Austria).

### Exploratory prospective cohort

In an exploratory analysis, we applied our AF prediction model to a small prospective cohort of patients enrolled as part of a separate clinical trial (NCT04529941). This included 32 patients with a history of symptomatic paroxysmal AF, normal LA size and left ventricular ejection fraction, and no history of heart failure, who underwent continuous remote telemetry monitoring for 7 days to quantify AF burden. Patients had a baseline TTE performed at the start of the study contemporaneously with remote monitoring initiation. We sought to determine whether our paroxysmal AF prediction model applied to these seemingly structurally normal TTEs could predict which patients would have significant AF. We used three previously described AF burden thresholds: 6 min, 1.5 h, and 24 h, all of which have been used to distinguish significantly different stroke risk categories^45–47^.

### Ensemble model

In order to understand whether TTEs and ECGs provide complementary information for predicting AF in patients with sinus rhythm, we created an ensemble model which gave a prediction based on a linear combination of the predictions from our TTE-based model as well as a previously published ECG-based model. The ECG-based model was originally trained for predicting AF within 31 days from a sinus rhythm ECG^11^.

We isolated a subset of our TTE validation and test cohorts that could be paired with pre-processed 12-lead sinus rhythm ECGs from our prior ECG-AF study within 90 days of each TTE. We applied min-max normalization to the TTE-based model predictions and then to the ECG-based model predictions to ensure that both model predictions ranged from 0 to 1. Using the validation TTE and ECG pairs, we used linear regression to derive the optimal linear weights for combining the TTE model and ECG model predictions. We then applied these weights to the test cohort TTE and ECG pairs to derive ensemble predictions. We then created ROC curves from the test cohort predictions comparing the performance of the ensemble predictions to those from the TTE and ECG models alone. We used the paired DeLong’s test for comparing the AUCs of the ROC curves.

### Model interpretability

We attempted to visualize which TTE structures the model focused on using multiple modern neural network interpretability methods, including Integrated Gradients and DeepLIFT^48^. These methods attempt to draw out signals of focus using backpropagation-based attribution algorithms.

This deep learning project accords with standards set forth by the MI-CLAIMS machine learning checklist (Supplementary Table 2)^49^.

### Reporting summary

Further information on research design is available in the Nature Research Reporting Summary linked to this article.

### Supplementary information

Supplemental material

Reporting Summary

## Data Availability

Due to patient privacy concerns, the data used in this study is available from the corresponding author upon request and after the establishment of data-sharing agreements between institutions.

## Code availability

The underlying code for this study is not publicly available but may be made available to qualified researchers on reasonable request from the corresponding author.
