# Supplementary material for: Deep learning evaluation of echocardiograms to identify occult atrial fibrillation
Source: NPJ Digit Med. 2024 Apr 13;7:96. doi: 10.1038/s41746-024-01090-z (PMC11016113; doi:10.1038/s41746-024-01090-z)
Supplement: Supplementary file 1 — Supplemental material [file 41746_2024_1090_MOESM1_ESM.pdf]

SUPPLEMENT

Supplementary Table 1. ICD-10 definitions for comorbidities

**Description**	**ICD-10 codes**
Atrial Fibrillation, Atrial Flutter	I48
Heart failure	I25.5, I50, I11.0, I13.2, I13.9, I42.0, I42.5-9, I43
Hypertension	I10, I11, I12, I13, I15
Prior CVA/TIA/TE	I63, I64.9, G45, I74, I26
Prior myocardial infarction	I21, I23
Peripheral arterial disease	I70, I71, I73.9
Diabetes	E10, E11
Smoker	F17.1, F17.29, F17.21, F17.20, Z86.43, Z87.891, Z71.6, Z72.0

Supplementary Figure 1. Deep learning model performance for predicting paroxysmal AF within +/- 365 days from TTEs in sinus rhythm.

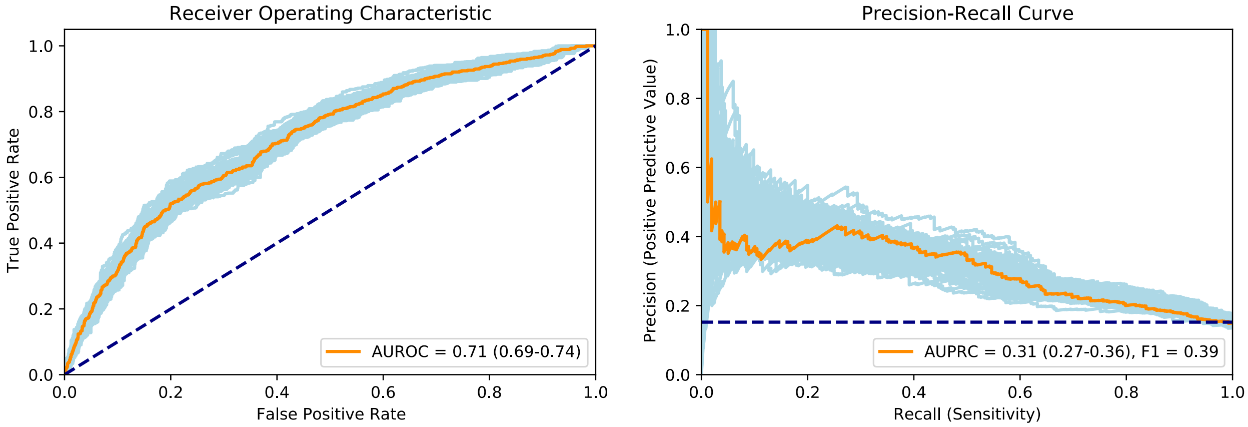

Supplementary Figure 2. Deep learning model performance for predicting history of AF in an external site TTE cohort

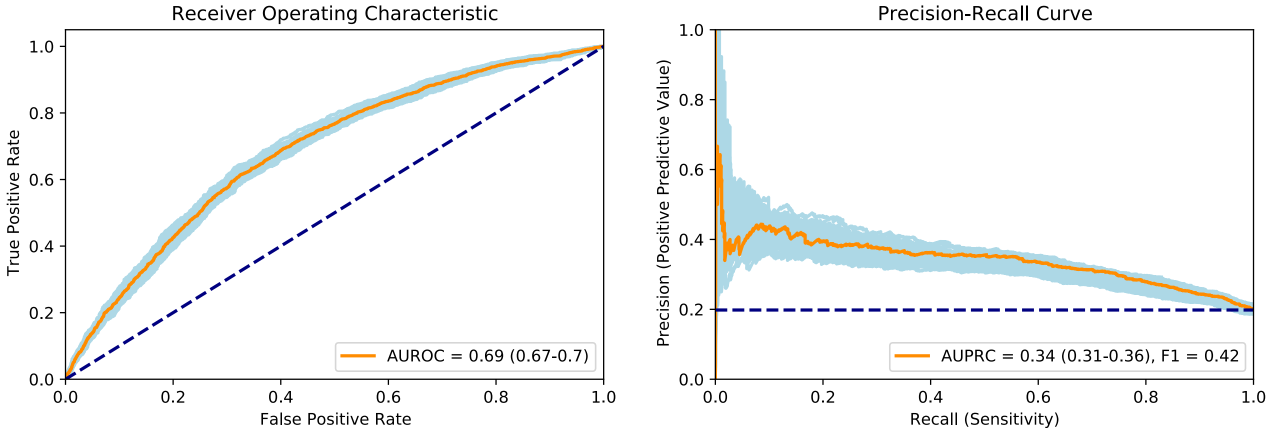

Supplementary Figure 3. AF prediction performance of an ensemble model using predictions from both TTEs and ECGs compared to models using either TTE or ECG alone.

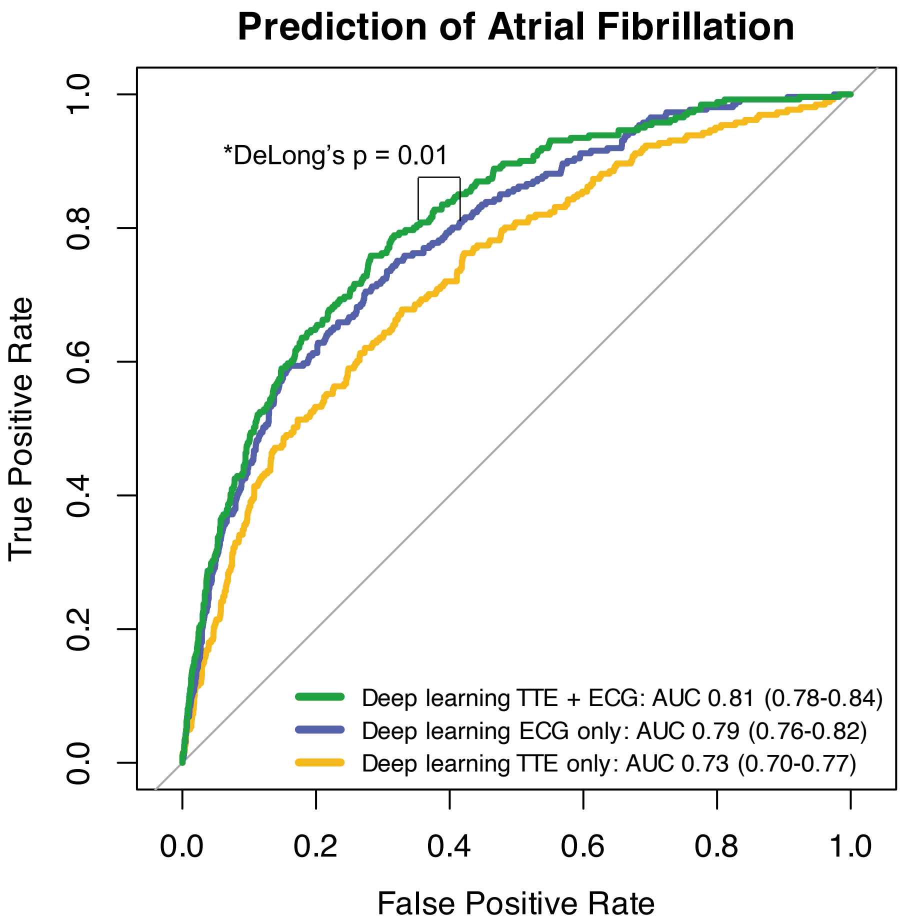

Supplementary Figure 4. Interpretability analysis using DeepLIFT.
Representative images from applying DeepLIFT, a modern backpropagation-based attribution method. Red highlights indicate areas of largest relative influence for the convolutional neural network.

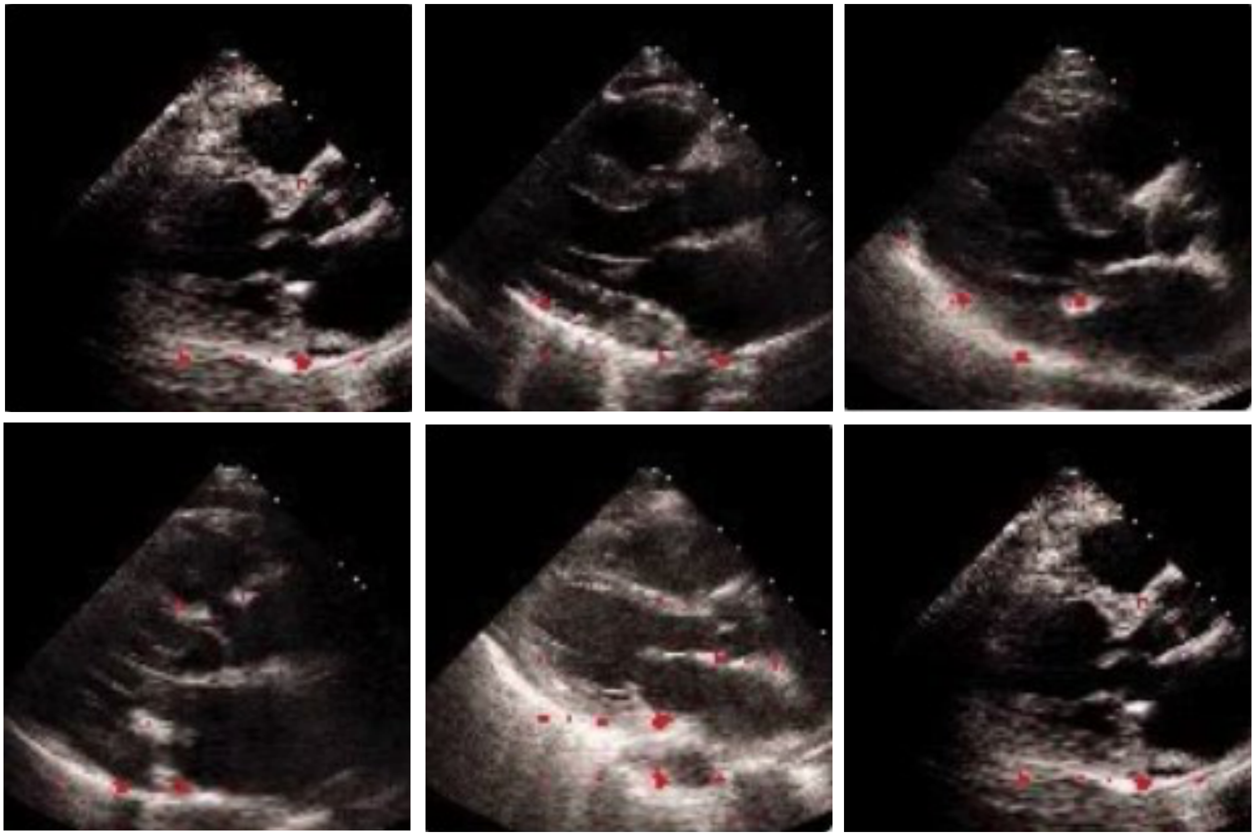

Supplementary Table 2. MI-CLAIMS checklist for machine learning study

**Before paper submission**			
**Study design (Part 1)**	**Completed:**	**page number**	**Notes**
The clinical problem in which the model will be employed is clearly detailed in the paper.	X	3	
The research question is clearly stated.	X	4	
The characteristics of the cohorts (training and test sets) are detailed in the text.	X	13	
The cohorts (training and test sets) are shown to be representative of real-world clinical settings.	X	4,5	Model was shown to generalize across patient subgroup and external populations. The cohort is enriched for AF patients, which has been further discussed in the limitations section.
The state-of-the-art solution used as a baseline for comparison has been identified and detailed.	X	5	
**Data and optimization (Parts 2, 3)**	**Completed:**	**page number**	**Notes**
The origin of the data is described and the original format is detailed in the paper.	X	11	
Transformations of the data before it is applied to the proposed model are described.	X	13	
The independence between training and test sets has been proven in the paper.	X	13	Training and test dataset splits were randomly assigned and did not have overlapping data.
Details on the models that were evaluated and the code developed to select the best model are provided.	X	14	
Is the input data type structured or unstructured?	☐ Structured	x Unstructured	
**Model performance (Part 4)**	**Completed:**	**page number**	**Notes**
The primary metric selected to evaluate algorithm performance (eg: AUC, F-score, etc) including the justification for selection, has been clearly stated.	X	15	
The primary metric selected to evaluate the clinical utility of the model (eg PPV, NNT, etc) including the justification for selection, has been clearly stated.	X	15	

The performance comparison between baseline and proposed model is presented with the appropriate statistical significance.	X	5	
**Model Examination (Parts 5)**	**Completed:**	**page number**	**Notes**
Examination Technique 1[a]	X	17	DeepLift and Integrated Gradients saliency mappings
Examination Technique 2[a]	X	4,5	Generalization further assessed by:<br>1. Patient demographic subgroup analyses<br>2. External cohort<br>3. Small prospective cohort
A discussion of the relevance of the examination results with respect to model/algorithm performance is presented.	X	8	
A discussion of the feasibility and significance of model interpretability at the case level if examination methods are uninterpretable is presented.	X	11	
A discussion of the reliability and robustness of the model as the underlying data distribution shifts is included.	X	5	
Common examination approaches based on study type:<br> For studies involving exclusively structured data coefficients and sensitivity analysis are often appropriate<br>* For studies involving unstructured data in the domains of image analysis or NLP: saliency maps (or equivalents) and sensitivity analysis are often appropriate			
**Reproducibility (Part 6): choose appropriate tier of transparency**			**Notes**
Tier 1: complete sharing of the code		☐	
Tier 2: allow a third party to evaluate the code for accuracy/fairness; share the results of this evaluation		X	
Tier 3: release of a virtual machine (binary) for running the code on new data without sharing its details		☐	
Tier 4: no sharing		☐	

PPV: Positive Predictive Value
NNT: Numbers Needed to Treat
[a] Common examination approaches based on study type: for studies involving exclusively structured data, coefficients and sensitivity analysis are often appropriate; for studies involving unstructured data in the domains of image analysis or natural language processing, saliency maps (or equivalents) and sensitivity analyses are often appropriate. Select 2 from this list or chose an appropriate technique, document each technique used on the appropriate line above.
