# Supplementary material for: Deep learning evaluation of echocardiograms to identify occult atrial fibrillation
Source: NPJ Digit Med. 2024 Apr 13;7:96. doi: 10.1038/s41746-024-01090-z (PMC11016113; doi:10.1038/s41746-024-01090-z)
Supplement: Supplementary file 2 — Reporting Summary [file 41746_2024_1090_MOESM2_ESM.pdf]

nature portfolio

Corresponding author(s): Neal Yuan
Last updated by author(s): Jan 29, 2024

Reporting Summary

Nature Portfolio wishes to improve the reproducibility of the work that we publish. This form provides structure for consistency and transparency in reporting. For further information on Nature Portfolio policies, see our Editorial Policies and the Editorial Policy Checklist.

Statistics

For all statistical analyses, confirm that the following items are present in the figure legend, table legend, main text, or Methods section.

n/a	Confirmed	
☐	☒	The exact sample size (*n*) for each experimental group/condition, given as a discrete number and unit of measurement
☐	☒	A statement on whether measurements were taken from distinct samples or whether the same sample was measured repeatedly
☒	☐	The statistical test(s) used AND whether they are one- or two-sided<br>*Only common tests should be described solely by name; describe more complex techniques in the Methods section.*
☐	☒	A description of all covariates tested
☒	☐	A description of any assumptions or corrections, such as tests of normality and adjustment for multiple comparisons
☒	☐	A full description of the statistical parameters including central tendency (e.g. means) or other basic estimates (e.g. regression coefficient) AND variation (e.g. standard deviation) or associated estimates of uncertainty (e.g. confidence intervals)
☒	☐	For null hypothesis testing, the test statistic (e.g. *F*, *t*, *r*) with confidence intervals, effect sizes, degrees of freedom and *P* value noted<br>*Give P values as exact values whenever suitable.*
☐	☐	For Bayesian analysis, information on the choice of priors and Markov chain Monte Carlo settings
☒	☐	For hierarchical and complex designs, identification of the appropriate level for tests and full reporting of outcomes
☒	☐	Estimates of effect sizes (e.g. Cohen's *d*, Pearson's *r*), indicating how they were calculated

Our web collection on statistics for biologists contains articles on many of the points above.

Software and code

Policy information about availability of computer code

Data collection	Echocardiogram videos were acquired using Philips EPIQ 7 or iE33 ultrasound machines.
Data analysis	All deep learning was performed using Python library PyTorch. We used the R2+1D convolutional neural network model. Additional data analysis was completed using R software (version 3.4.1, Vienna, Austria)

For manuscripts utilizing custom algorithms or software that are central to the research but not yet described in published literature, software must be made available to editors and reviewers. We strongly encourage code deposition in a community repository (e.g. GitHub). See the Nature Portfolio guidelines for submitting code & software for further information.

Data

Policy information about availability of data

All manuscripts must include a data availability statement. This statement should provide the following information, where applicable:

- Accession codes, unique identifiers, or web links for publicly available datasets
- A description of any restrictions on data availability
- For clinical datasets or third party data, please ensure that the statement adheres to our policy

DATA AVAILABILITY
Due to patient privacy concerns, the data used in this study is available from the corresponding author upon request and after establishment of data sharing agreements between institutions.

CODE AVAILABILITY
The underlying code for this study is not publicly available but may be made available to qualified researchers on reasonable request from the corresponding author.

Research involving human participants, their data, or biological material

Policy information about studies with human participants or human data. See also policy information about sex, gender (identity/presentation), and sexual orientation and race, ethnicity and racism.

Reporting on sex and gender	We used sex based analysis given this is the demographic available in the electronic records.
Reporting on race, ethnicity, or other socially relevant groupings	We used standard race/ethnicity groupings that were already pre-categorized in the electronic records: American Indian, Asian, Black, Hispanic, White, Pacific Islander, Other.
Population characteristics	These are displayed in our Table 1.
Recruitment	We did not recruit patients for this study
Ethics oversight	The study was approved by the IRB at Cedars-Sinai Medical Center.

Note that full information on the approval of the study protocol must also be provided in the manuscript.

Field-specific reporting

Please select the one below that is the best fit for your research. If you are not sure, read the appropriate sections before making your selection.

☒ Life sciences ☐ Behavioural & social sciences ☐ Ecological, evolutionary & environmental sciences

For a reference copy of the document with all sections, see nature.com/documents/nr-reporting-summary-flat.pdf

Life sciences study design

All studies must disclose on these points even when the disclosure is negative.

Sample size	No sample size calculation was performed.
Data exclusions	We included only those TTEs that were in AF (atrial fibrillation or atrial flutter) or sinus rhythm per the TTE report and by the absence or presence of an identified mitral A wave Doppler velocity. In order to improve label accuracy, TTEs in sinus rhythm were additionally required to have a sinus rhythm ECG within 24 hours (Figure 1).
Replication	Our results were replicated in an external dataset and a small prospective study.
Randomization	This was not a randomized study
Blinding	This was not a blinded study

Reporting for specific materials, systems and methods

We require information from authors about some types of materials, experimental systems and methods used in many studies. Here, indicate whether each material, system or method listed is relevant to your study. If you are not sure if a list item applies to your research, read the appropriate section before selecting a response.

Materials & experimental systems

n/a	Involved in the study	
☒	☐	Antibodies
☒	☐	Eukaryotic cell lines
☒	☐	Palaeontology and archaeology
☒	☐	Animals and other organisms
☐	☒	Clinical data
☒	☐	Dual use research of concern
☒	☐	Plants

Methods

n/a	Involved in the study	
☒	☐	ChIP-seq
☒	☐	Flow cytometry
☒	☐	MRI-based neuroimaging

Clinical data

Policy information about clinical studies

All manuscripts should comply with the ICMJE guidelines for publication of clinical research and a completed CONSORT checklist must be included with all submissions.

Clinical trial registration	NA, not a trial (CONSORT checklist therefore also not applicable). We have filled out a machine learning checklist MI-CLAIMS instead and have included it with our manuscript.
Study protocol	NA
Data collection	We identified all TTEs performed between 6/2004 and 6/2021 at Cedars-Sinai Medical Center.
Outcomes	Outcomes were prediction performance for predicting active AF and hidden AF from TTEs

Plants

Seed stocks	NA
Novel plant genotypes	NA
Authentication	NA
